# Radiomic signatures from baseline CT predict chemotherapy response in unresectable colorectal liver metastases

**DOI:** 10.1117/1.JMI.13.1.014505

**Published:** 2026-01-13

**Authors:** Mane Piliposyan, Jacob J. Peoples, Mohammad Hamghalam, Ramtin Mojtahedi, Kaitlyn Kobayashi, E. Claire Bunker, Natalie Gangai, Hyunseon C. Kang, Yun Shin Chun, Christian Muise, Richard K. G. Do, Amber L. Simpson

**Affiliations:** a Queen’s University, School of Computing, Kingston, Ontario, Canada; b Queen’s University, Department of Biomedical and Molecular Sciences, Kingston, Ontario, Canada; c Memorial Sloan Kettering Cancer Center, Department of Radiology, New York, United States; d University of Texas MD Anderson Cancer Center, Department of Radiology, Houston, Texas, United States; e University of Texas MD Anderson Cancer Center, Department of Surgery, Houston, Texas, United States

**Keywords:** quantitative image analysis, Response Evaluation Criteria in Solid Tumours, colorectal liver metastases, treatment response prediction, radiomics, radiomic signatures

## Abstract

**Purpose:**

Colorectal cancer is the third most common cancer globally, with a high mortality rate due to metastatic progression, particularly in the liver. Surgical resection remains the main curative treatment, but only a small subset of patients is eligible for surgery at diagnosis. For patients with initially unresectable colorectal liver metastases (CRLM), neoadjuvant chemotherapy can downstage tumors, potentially making surgery feasible. We investigate whether radiomic signatures—quantitative imaging biomarkers derived from baseline computed tomography (CT) scans—can noninvasively predict chemotherapy response in patients with unresectable CRLM, offering a pathway toward personalized treatment planning.

**Approach:**

We used radiomics combined with a stacking classifier (SC) to predict treatment outcome. Baseline CT imaging data from 355 patients with initially unresectable CRLM were analyzed using two regions of interest (ROIs) separately (all tumors in the liver and the largest tumor by volume). From each ROI, 107 radiomic features were extracted. The dataset was split into training and testing sets, and multiple machine learning models were trained and integrated via stacking to enhance prediction. Logistic regression coefficients were used to derive radiomic signatures.

**Results:**

The SC achieved strong predictive performance, with an area under the receiver operating characteristic curve of up to 0.77 for response prediction. Logistic regression identified 12 and 7 predictive features for treatment response in all tumors and the largest tumor ROIs, respectively.

**Conclusion:**

Our findings demonstrate that radiomic features from baseline CT scans can serve as robust, interpretable biomarkers for predicting chemotherapy response, offering insights to guide personalized treatment in unresectable CRLM.

## 1 Introduction

Colorectal cancer is the third most common cancer worldwide, accounting for ∼10% of cases^1^ and 9.4% of cancer-related deaths.^2^ Approximately 25% of the patients have colorectal liver metastases (CRLM) at diagnosis, and 50% will develop CRLM over time.^3^ Although some newer treatment regimes, such as immunotherapy, have demonstrated remarkable effectiveness in treating patients with CRLM,^3^ surgical resection remains the primary treatment for achieving a full recovery with 5-year survival rates exceeding 50%.^4^ However, only 25% of patients are eligible for resection upon diagnosis.^4^ For 24% to 52% of patients, neoadjuvant chemotherapy (NAC) can downstage disease, shrinking tumors to enable potentially curative resection. ^5^ NAC is also known to lower the chance of disease recurrence.^6^ With surgery, the predicted 5-year survival rate is 63% to 72%, offering promise to patients with an aggressive disease.^7^ Predicting whether a patient will respond to chemotherapy is crucial so that patients can avoid a potentially toxic therapy^8^ or be given another potentially life-saving therapy earlier. In addition, identifying patients most or least likely to respond to chemotherapy would allow a targeted approach for downstaging cancer and improve patient survival of this deadly disease.

**Fig. 1:**
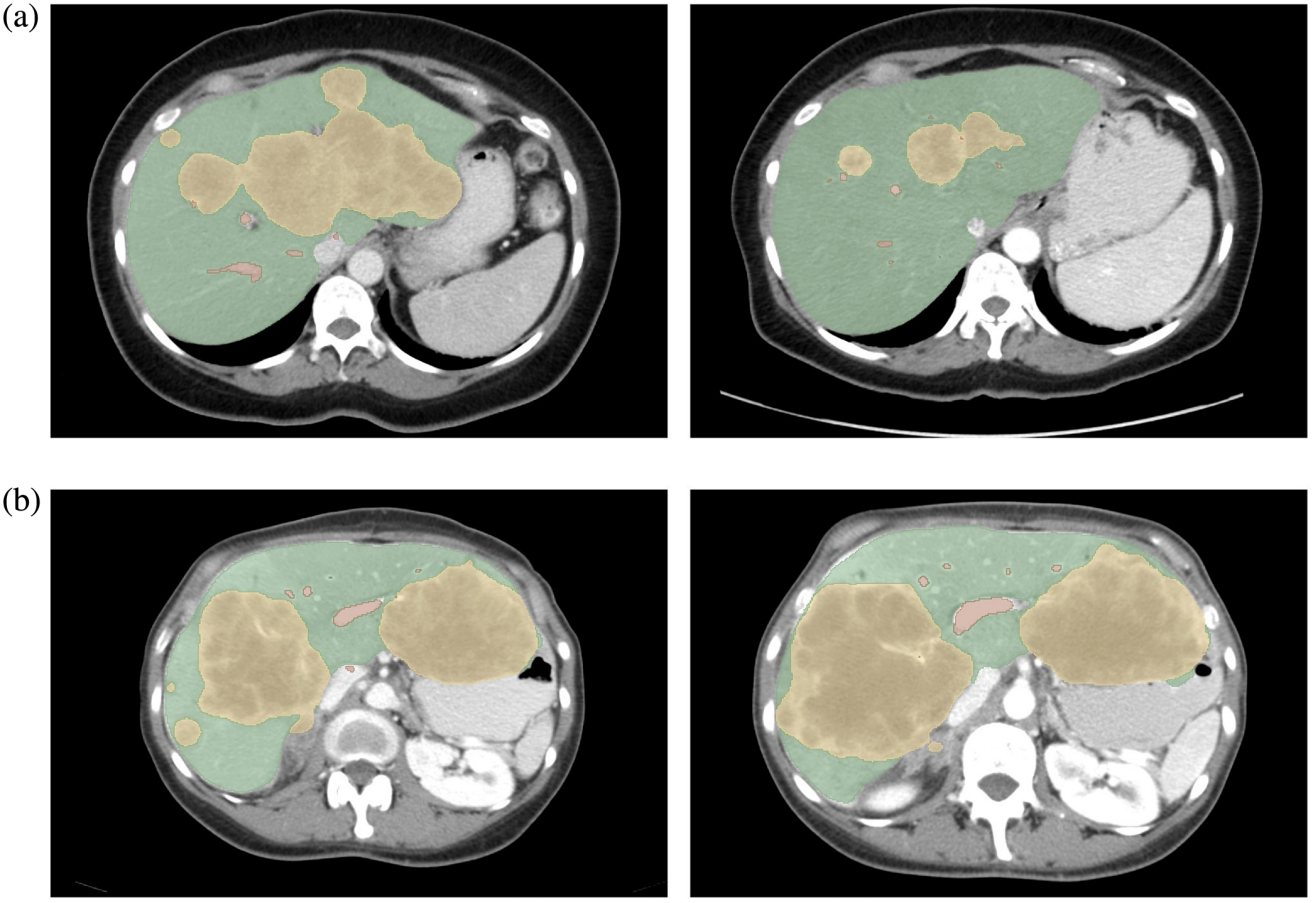
CT scans of two patients before and after NAC treatment showing response and nonresponse (liver = green, vessels = red, and tumors = yellow): (a) patient who responded to NAC and (b) patient who did not respond to NAC.

Computed tomography (CT) imaging plays a crucial role in determining the staging of CRLM. Response to chemotherapy varies due to tumor heterogeneity.^9^ Figure 1 shows examples before (left column images) and after (right column images) NAC treatment of a patient who responds to chemotherapy (a) compared with the one who does not (b) in CT imaging.

Radiomics, the high-throughput extraction of quantitative features from medical images that may not be visible to the naked eye, enables deeper insights from routine scans.^10^ The pixels in a CT scan are represented in terms of Hounsfield units (HUs), which measure the density of tissues. Denser tissues have higher HU values and appear whiter on the scans, whereas less dense tissues are typically darker. This numerical representation enables us to quantify tissue properties and extract hidden patterns that can provide insights into the underlying conditions that are otherwise unavailable in traditional clinical reports. Radiomic models have been successful in the prediction of response in lung and other cancers.^11^ From the pool of radiomic features, it is possible to define a reproducible radiomic signature composed of those features most strongly associated with clinical outcomes. Such signatures have shown promise in prognostic modelling across various cancers, including survival prediction and tumor progression.^12^^–^^15^

The objective of this study is to evaluate the predictive power of a single pre-treatment CT scan in determining whether or not patients with initially unresectable CRLM will respond to chemotherapy. Creasy et al.^16^ showed that volumetric response could be predicted using a regression model trained on radiomic features extracted from a single pre-treatment scan prior to the initiation of treatment; however, the study was limited in sample size, a gap we sought to address in this paper. We introduce noninvasive radiomic models on a large cohort of patients specifically designed for *a priori* prediction of chemotherapy response in patients with initially unresectable CRLM. By leveraging baseline CT imaging, our framework addresses a critical gap in personalized treatment planning for this high-risk population.

For each patient, the evaluation of tumor response was based on the Response Evaluation Criteria in Solid Tumours (RECIST 1.1).^17^ Despite being the standard for clinical trials, RECIST measurements depend on uni-dimensional measurements, which might not precisely represent the total tumor burden. Recent technical advancements have enabled the development of volume-based tumor measurements, which are notably more sensitive to changes in tumor size and are more reproducible and reliable than traditional methods. In this study, response is quantified using volumetric criteria aligned with RECIST 1.1, guided by Winter et al.^18^; full details are in Sec. 2.

## 2 Materials and Methods

The study was approved by the Institutional Review Board at Memorial Sloan Kettering (MSK) as well as the Research Ethics Board of Queen’s University. Patients included in the study were diagnosed with initially unresectable CRLM and received chemotherapy at MSK. Our study further required that all patients undergo a pre-treatment and post-treatment contrast-enhanced CT scan in the portal venous phase, with the post-treatment scan occurring 8 weeks after chemotherapy. The initial query returned 615 patients with pre-treatment and post-treatment CT scans between 2009 and 2020.

**Fig. 2:**
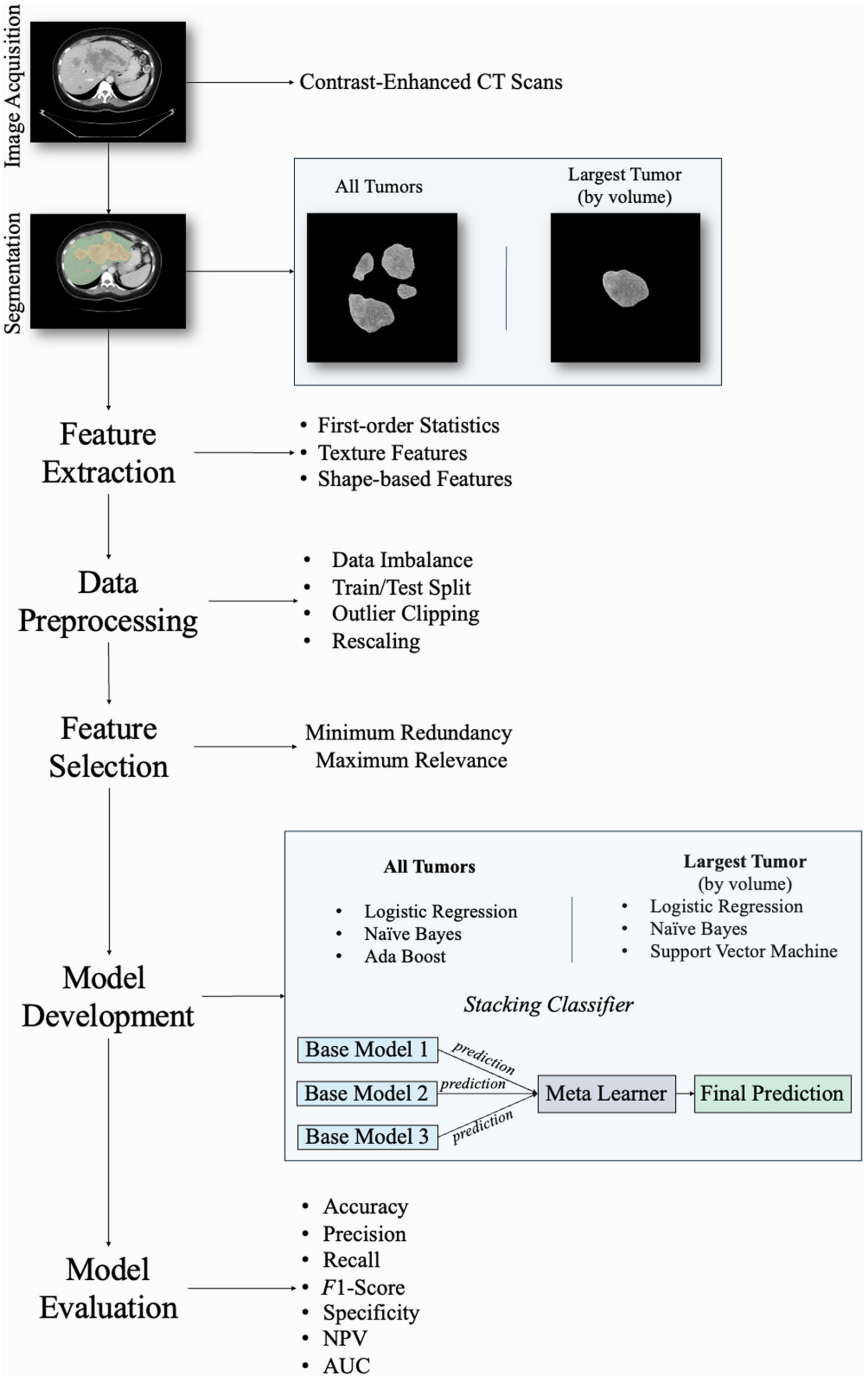
Overview of the approach. Negative predictive value (NPV) and area under the curve (AUC).

Figure 2 provides a summary of the pipeline. Each step in the workflow is described in detail in the following sections.

### 2.1 Response Categories

Winter et al.^18^ conducted a retrospective study aimed at deriving volumetric thresholds that correspond to the established RECIST 1.1 guidelines for evaluating tumor response. Researchers examined up to five metastases per patient, comparing changes in diameter and volume. The results showed that volumetric thresholds of −65% for partial response (tumor shrinkage) and +65% for progressive disease (tumor growth) closely aligned with the RECIST 1.1 unidimensional thresholds of −30% and +20%, respectively. Validation showed high concordance between RECIST assessments and volumetric measurements, confirming the reliability of the new criteria for classifying tumor response and progression.

Under this framework, four categories are defined, corresponding to the RECIST categories: complete response (CR) (total tumor disappearannce), partial response (PR) (>65% decrease in volume), stable disease (SD) (tumor volume changes by less than 65% in either direction), and progressive disease (PD) (tumor volume growth >65%). To simplify our analysis, we grouped patients into only two categories: responders (either complete or partial response) or nonresponders (progressive disease or stable disease).

### 2.2 Image Acquisition

Contrast-enhanced abdominal scans were acquired during the portal venous phase (80 s post-injection) using standardized clinical protocols. All imaging was acquired using multidetector CT scanners (Discovery, LightSpeed, Revolution series; GE Healthcare, Madison, Wisconsin, United States), configured with 4 to 128 detector rows, detector widths of 0.6 to 1.25 mm, and total collimation widths of 10 to 80 mm. Tube voltage was set to 120 to 140 kVp, with tube current either fixed [median 220 mA, interquartile range (IQR): 219 to 379 mA] or modulated using GE smart mA (noise index 8 to 20, mA range 220 to 380). Gantry rotation times were predominantly 0.7 to 0.8 s, and pitch factors were most frequently 0.984. Reconstruction utilized a GE standard soft-tissue kernel, with slice thicknesses of 5.0 mm in the majority of cases (others: 2.5 mm, 3.75 mm, or sub-millimeter). Adaptive statistical iterative reconstruction (ASiR) was applied in most studies, primarily at a level of 20%.

### 2.3 Image Segmentation

**Fig. 3:**
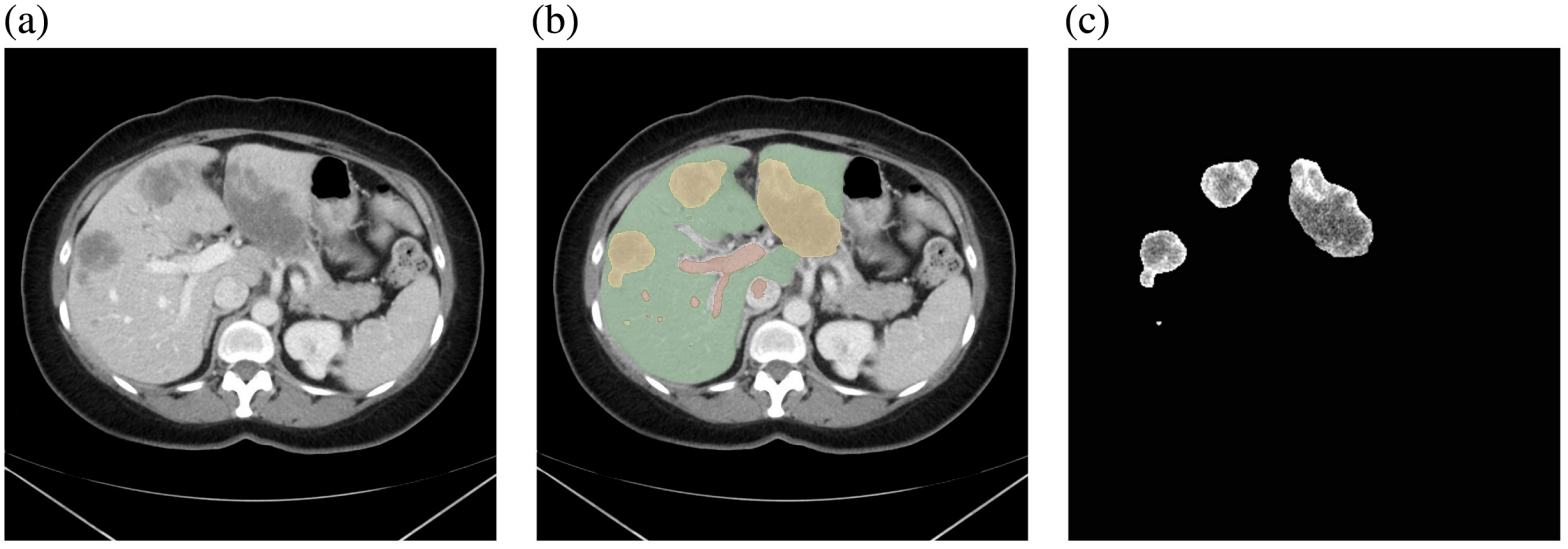
Example of liver segmentation and ROIs used in analysis: original CT scan (a), overlaid with segmentation mask (b) with liver parenchyma in green, tumor regions in yellow, and vascular structures in red, and all tumor region ROI (c). The largest tumor ROI (not shown) was also used.

A 3D U-Net structure^19^ was used for segmentation of the liver parenchyma, vessels, and tumors in 3D abdominal scans. Initially trained on 197 labelled datasets,^20^ the model was iteratively refitted on 186 unseen samples with ground truth segmentations reviewed by a radiologist. Unseen samples were added in small batches until the model performance began to plateau, achieving clinically acceptable Dice scores of ∼0.97 for the liver parenchyma and 0.76 for tumors.^21^ Figure 3(b) illustrates the model’s ability to accurately segment the liver parenchyma (green), tumors (yellow), and vessels (red). In comparison with recent benchmark results reported by nnU-Net Revisited,^22^ which found average liver + tumor Dice scores of 82.34% for CNN-based, 79.10% for transformer-based, and 80.40% for Mamba-based models on the LiTS^23^ dataset, our model achieved a higher average combined Dice of 86.50% (liver ≈0.97; tumor ≈0.76), noting that our results are based on a larger, multi-institutional dataset with different imaging characteristics.

For this study, two regions of interest (ROIs) were defined: the all tumor region and the largest tumor. All tumors include all tumor tissue within the liver, capturing the overall burden of the disease. Sub-centimeter lesions are retained, and in every case, the cumulative sum of lesion diameters exceeds 1 cm. The largest tumor focuses on the single largest lesion by volume, allowing for the detailed examination of the most significant area of the disease. In our cohort, this lesion was always ≥1  cm.

### 2.4 Feature Extraction

Radiomic features were extracted from all tumors and the largest tumor ROIs. Features were extracted in 3D, after preprocessing images to 1×1×1  mm isotropic voxels, using a discretization bin width of 25 Hounsfield units. In total, 107 default features were extracted from these regions, including first-order statistics (18), shape-based (14), gray level co-occurrence matrix (GLCM) features (24), gray level run length matrix (GLRLM) features (16), gray level size zone matrix (GLSZM) features (16), gray level dependence matrix (GLDM) (14), and neighborhood gray tone difference matrix (NGTDM) features (5).

### 2.5 Data Preprocessing

First, data imbalance was addressed by performing random under-sampling of the majority class to balance the data set between responders and nonresponders. After this, the data were split into training and testing sets using an 80 to 20 split, using stratified sampling to maintain the class distribution within both sets. The same subsets were then used for both regions of interest to ensure consistency in model evaluation.

To prepare the dataset for analysis, outlier clipping and rescaling were performed to improve data quality and optimize model performance. All the steps described below were done for both ROIs independently. Outliers were managed using the IQR.^24^ This involved calculating the lower (25th percentile) and upper (75th percentile) quartiles for each feature within the training set and defining boundaries as below:

upper boundary=Q1−k×IQR,lower boundary=Q3+k×IQR,

where Q1 and Q3 are the 25th and 75th quartiles, respectively, IQR=Q3−Q1, and k is the tolerance level. k was set to 1. Data points falling outside of these ranges were clipped to the respective lower and upper boundaries for both training and testing sets. Finally, features were rescaled based on the range observed in the training set to the range [−1,1] using the MinMaxScaler class in scikit-learn^25^ to ensure consistency across features. For both preprocessing steps, all parameters (clipping bounds and min/max) were estimated exclusively from the training set and applied unchanged to the test set, preventing information leakage.

### 2.6 Feature Selection

Feature selection was conducted using the minimum redundancy maximum relevance (mRMR)^26^ method to identify the most relevant features for the classification task. This approach ensured that the selected features were both highly relevant to the target variable and minimally redundant among themselves, thereby enhancing the models’ predictive power. The number of selected features was treated as a hyperparameter, and experiments were conducted until an acceptable model performance was reached. Ultimately, 20 features were determined to provide the optimal balance.

As shown in Algorithm 1, the selection process starts with picking the most relevant feature from the dataset with continuous features, and the algorithm selects the remaining features considering both relevance and redundancy.

**Algorithm 1:** mRMR selection.

**Require:** $X=\{X_{1},X_{2},\ldots ,X_{n}\}$ ▹Set of continuous features
1: Y ▹Categorical target variable
2: k ▹Number of features to select
**Ensure:** $S=\{S_{1},S_{2},\ldots ,S_{k}\}$ ▹Selected feature subset
3: S←Ø ▹Initialize selected feature set
▹Compute relevance for each feature with the target
4: **for** each $X_{i}\in X$ **do**
5: $\mathrm{relevance}[X_{i}]\leftarrow F-\mathrm{statistics}(X_{i},Y)$
6: **end for**
▹Select the feature with maximum relevance
7: $X_{\mathrm{selected}}\leftarrow \arg\max_{X_{i}\in X}\mathrm{relevance}[X_{i}]$
8: $S\leftarrow \{X_{\mathrm{selected}}\}$
▹Iteratively select remaining features
9: **while** |S|<k **do**
10: **for** each $X_{j}\in X\setminus S$ **do**
11: redundancy_sum←0
12: **for** each $S_{i}\in S$ **do**
13: $\mathrm{redundancy}\_\mathrm{sum}\leftarrow \mathrm{redundancy}\_\mathrm{sum}+\mathrm{Pearson}-\mathrm{Correlation}(X_{j},S_{i})$
14: **end for**
15: $\mathrm{score}[X_{j}]\leftarrow \mathrm{relevance}[X_{j}]-(\frac{\mathrm{redundancy}\_\mathrm{sum}}{\left|S\right|})$
16: **end for**
17: $X_{\mathrm{selected}}\leftarrow \arg\max_{X_{j}\in X\setminus S}\mathrm{score}[X_{j}]$
18: $S\leftarrow S\cup \{X_{\mathrm{selected}}\}$
19: **end while**
20: **return** S

### 2.7 Model Development

The final set of selected features was used to train different traditional machine learning (ML) models, such as logistic regression (LR), decision trees, random forest, support vector machine (SVM), K-nearest neighbors, AdaBoost (ADA), and naive Bayes (NB). A grid search was conducted for models’ hyperparameter tuning, and the three best-performing configurations were selected separately for each ROI.

**Fig. 4:**
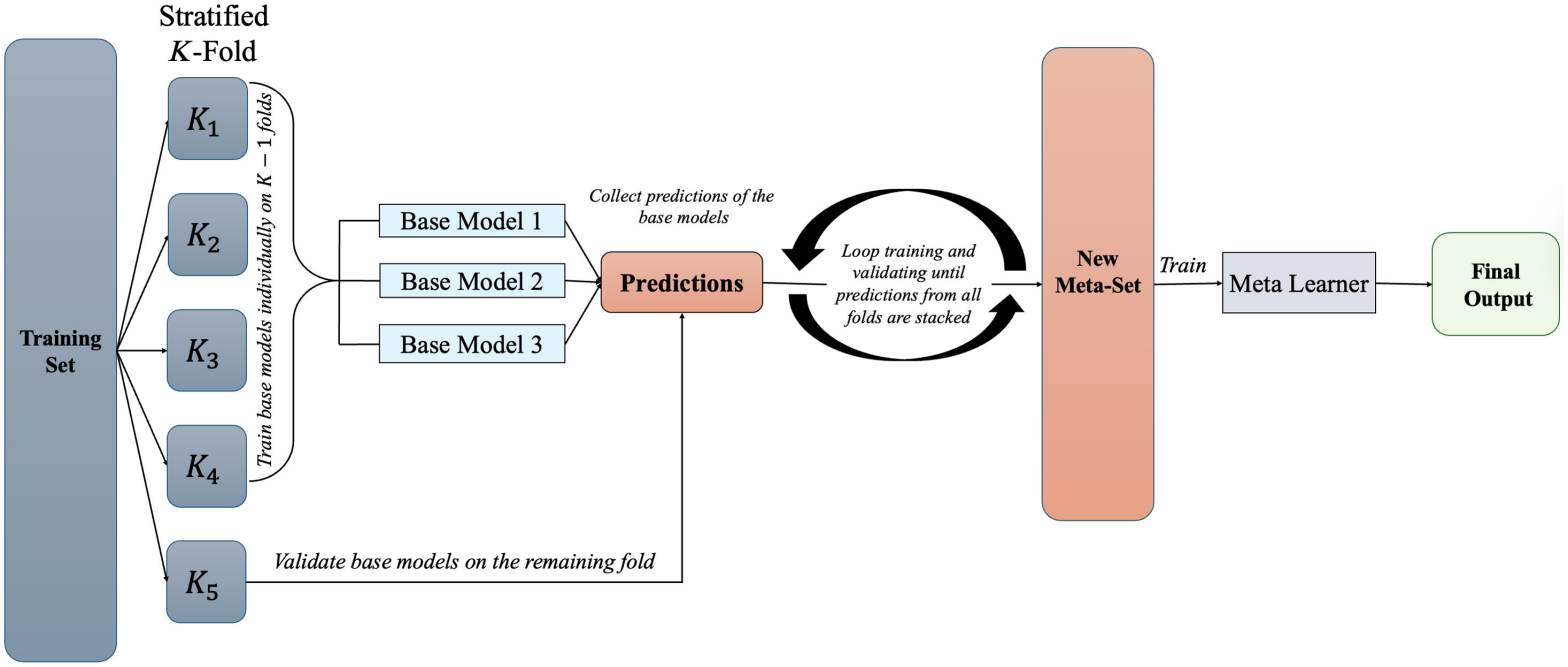
Training process of the SC that combines top-performing models into a meta learner.

To further enhance predictive power, we implemented a stacking classifier (SC) to combine the strengths of the best-performing models. The training process is illustrated in Fig. 4. This technique, also known as super learner, combines the predictions made by individual models and uses a secondary classifier, often called a meta-learner, to produce the final prediction.^27^ For this step, we used LR, which is the default model. To train the SC, a stratified fivefold cross-validation strategy is employed on the training dataset. In each fold, the base models were trained on four subsets and validated on the remaining one. Predictions from the validation folds were collected and concatenated to form a new meta-features set, ensuring that predictions were available for the entire training data without introducing data leakage. This meta-feature set was then used to train a meta-learner, which learned how to effectively combine the outputs of the base models. Finally, the base models were retrained on the entire training dataset to maximize their learning capacity before being used alongside the trained meta-learner for final predictions.

### 2.8 Evaluation

To evaluate the performance of the models, several metrics were used, including accuracy, precision, recall, F1-score, specificity, negative predictive value (NPV), and an area under the curve (AUC). These metrics provide a comprehensive understanding of the model’s ability to distinguish between responders and non-responders across the different ROIs.

For each model, we evaluated performance on the test set via bootstrapping (1000 resamples with replacement at the patient level). For each resample, predictions were obtained from the trained model, and metrics were computed. We report the bootstrap mean for each metric, together with 95% percentile confidence intervals (2.5th to 97.5th percentiles) from the bootstrap distribution.

To further gain insights into the relationship between radiomic model predictions and the patient response category, radiomic scores for each patient were derived, based on the LR model, by computing the dot product between the learned feature coefficients and the corresponding feature values for each patient in both ROIs, as shown in Eq. (1),

$${\mathrm{Score}}_{\mathrm{patient}}=\overset{n}{\underset{i=1}{\sum }}({\mathrm{coef}}_{{\mathrm{feature}}_{i}}\cdot {\mathrm{feature}}_{i,\mathrm{patient}}).$$ (1)

## 3 Results

### 3.1 Exclusion Criterias

**Fig. 5:**
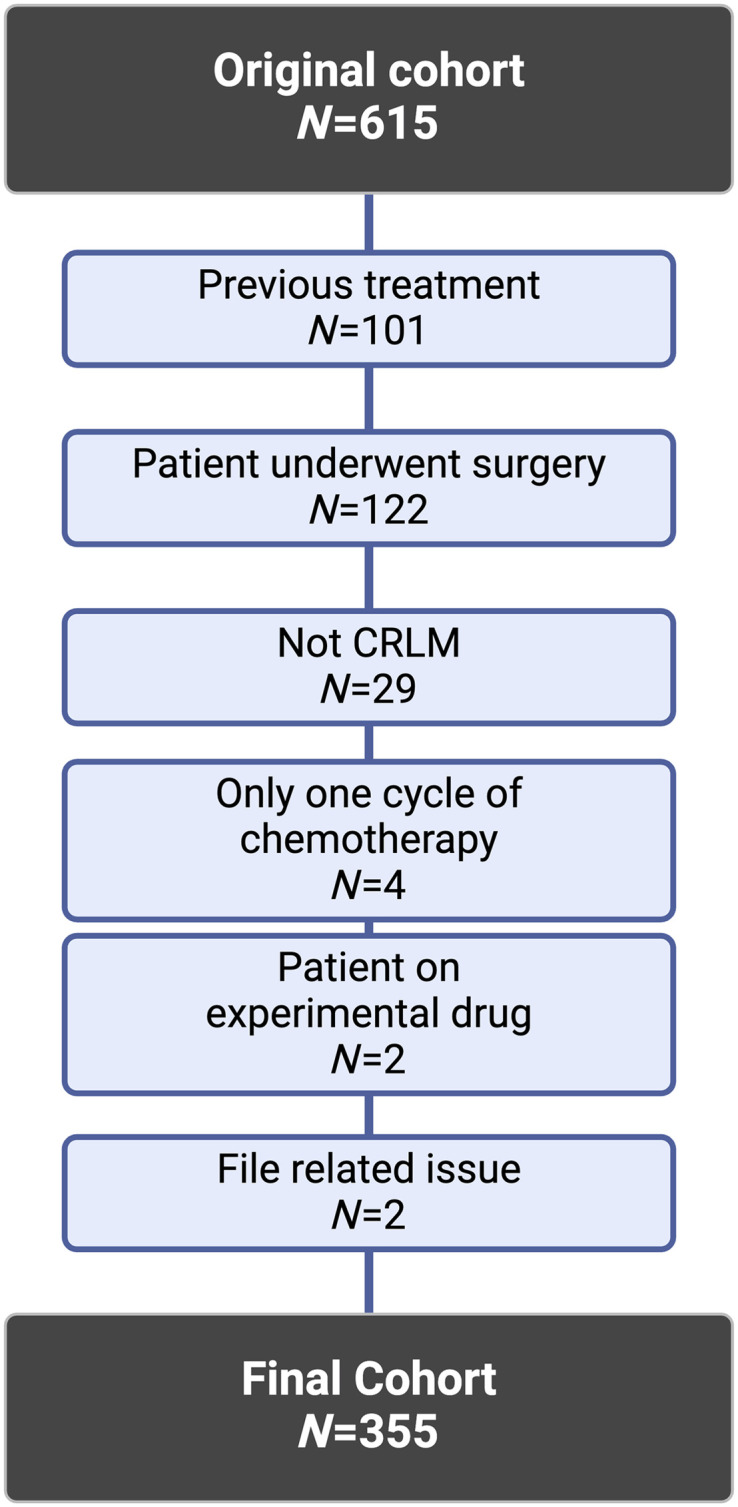
Flowchart of patient inclusion and exclusion criteria.

From the initial 615 identified patients, a total of 355 patients remained in the study after applying all necessary exclusions (see Fig. 5). Of these, CR was observed in four patients, PR in 152, SD in 174, and PD in 25 cases. After grouping the categories, we obtained a total of 156 responders (43.94%) and 199 non-responders (56.06%). After random undersampling to balance the classes, we were left with 312 patients, split into 241 for training and 58 for testing.

### 3.2 Model Performance

The detailed breakdown of the selected features, organized by feature category for each ROI, is provided in Table 1. Table 2 summarizes the performance metrics of the classifiers for the two ROIs. The results show that the models performed better when using all tumors, as opposed to the largest tumor.

**Table 1:** Selected features grouped by feature category. Informational measure of correlation (IMC) and maximal correlation coefficient (MCC).

Feature category	All tumor features	Largest tumor features
Shape	Elongation	Elongation
	Sphericity	Flatness
	Major axis length	
First-order statistics	Kurtosis	Kurtosis
	Median	Median
	Energy	Total energy
	Robust mean absolute deviation	Root mean squared
		10th percentile
		Mean
GLCM	Cluster shade	Cluster shade
	IMC1	IMC1
	MCC	MCC
		Joint energy
		Maximum probability
GLRLM	Low gray level run emphasis	Short run low gray level emphasis
		Run length nonuniformity
GLSZM	Gray level nonuniformity normalized	Gray level nonuniformity normalized
	Size zone nonuniformity normalized	Size zone nonuniformity normalized
	Low gray level zone emphasis	Low gray level zone emphasis
	Small area high gray level emphasis	Small area emphasis
GLDM	Low gray level emphasis	Low gray level emphasis
	Small dependence high gray level emphasis	
	Small dependence low gray level emphasis	
	Large dependence high gray level emphasis	
NGTDM	Contrast	Contrast

**Table 2:** Performance metrics for selected classifiers derived from 1000 iterations of bootstrapping with 95% CI on the testing set.

ROI	Model	Accuracy	Precision	Recall	F1-score	Specificity	NPV	AUC
All tumors	LR	0.707 (0.586, 0.810)	0.697 (0.533, 0.852)	0.728 (0.560, 0.885)	0.709 (0.571, 0.820)	0.687 (0.516, 0.852)	0.718 (0.531, 0.882)	0.775 (0.645, 0.887)
	NB	0.742 (0.621, 0.845)	0.715 (0.553, 0.861)	0.799 (0.654, 0.929)	0.752 (0.621, 0.862)	0.685 (0.500, 0.849)	0.774 (0.583, 0.929)	0.757 (0.615, 0.879)
	Ada	0.672 (0.552, 0.776)	0.652 (0.485, 0.807)	0.729 (0.567, 0.889)	0.685 (0.540, 0.806)	0.616 (0.435, 0.783)	0.697 (0.517, 0.867)	0.693 (0.548, 0.821)
	SC	0.743 (0.621, 0.845)	0.748 (0.586, 0.900)	0.728 (0.560, 0.885)	0.735 (0.600, 0.846)	0.757 (0.594, 0.903)	0.738 (0.556, 0.893)	0.775 (0.645, 0.887)
Largest tumor	LR	0.656 (0.534, 0.776)	0.654 (0.471, 0.821)	0.657 (0.469, 0.828)	0.651 (0.491, 0.780)	0.656 (0.471, 0.833)	0.659 (0.478, 0.829)	0.725 (0.579, 0.852)
	NB	0.726 (0.620, 0.845)	0.696 (0.531, 0.852)	0.797 (0.647, 0.929)	0.740 (0.612, 0.853)	0.655 (0.464, 0.828)	0.765 (0.586, 0.920)	0.744 (0.615, 0.862)
	SVM	0.692 (0.569, 0.810)	0.656 (0.486, 0.812)	0.796 (0.640, 0.929)	0.716 (0.571, 0.831)	0.588 (0.400, 0.769)	0.745 (0.556, 0.909)	0.723 (0.585, 0.860)
	SC	0.708 (0.586, 0.828)	0.675 (0.514, 0.833)	0.795 (0.633, 0.926)	0.727 (0.592, 0.849)	0.622 (0.440, 0.786)	0.754 (0.577, 0.913)	0.724 (0.582, 0.852)

Among all the models, the SC demonstrated the best overall performance, achieving an AUC of 0.775 for all tumor ROI, indicating a strong ability to distinguish between responders and nonresponders. Furthermore, an F1 score of 0.735 reflects the best balance between precision and recall compared with the other models.

LR showed moderate performance, with an AUC of 0.775, matching that of the SC. However, its precision and specificity were lower at 0.697 and 0.728, respectively. Although the model’s performance was still satisfactory—especially in terms of identifying true responders (recall of 0.728) and nonresponders (NPV of 0.718)—it did not achieve the same level of balance as SC.

The NB classifier also performed well, particularly in terms of recall, achieving the highest value at 0.799, making it the most effective model in correctly identifying the true responders. This, however, came with a slightly lower precision of 0.715, suggesting a higher rate of false positives compared to the SC. Nevertheless, NB’s AUC of 0.757 supports its effectiveness in classifying responders.

The Ada model displayed the lowest performance among the models, with an AUC of 0.693 and an F1 score of 0.685. Although its recall of almost 73% is comparable to the other models, its precision was notably lower at only around 65.2%, indicating more false positives compared with the other classifiers. In addition, Ada’s specificity of 0.616 and NPV of 0.697 further highlight its limitations in reliably distinguishing between responders and nonresponders.

For the largest tumor, NB, SVM, and SC models demonstrated similar trends. All three classifiers excelled at identifying true positives, with recall rates nearing 80%. However, their precision remained below 70%, reflecting a trade-off with a higher false positive rate, meaning they struggled to accurately identify nonresponders.

Although LR had lower performance metrics for the largest tumor ROI when compared with all tumors ROI, it still demonstrated balanced performance overall, with precision, recall, and F1 scores being at 0.654, 0.657, and 0.651, respectively. With an AUC of 0.725, LR demonstrated reasonable reliability, offering a more consistent balance between identifying both responders and nonresponders than the other models.

### 3.3 Radiomic Score

**Fig. 6:**
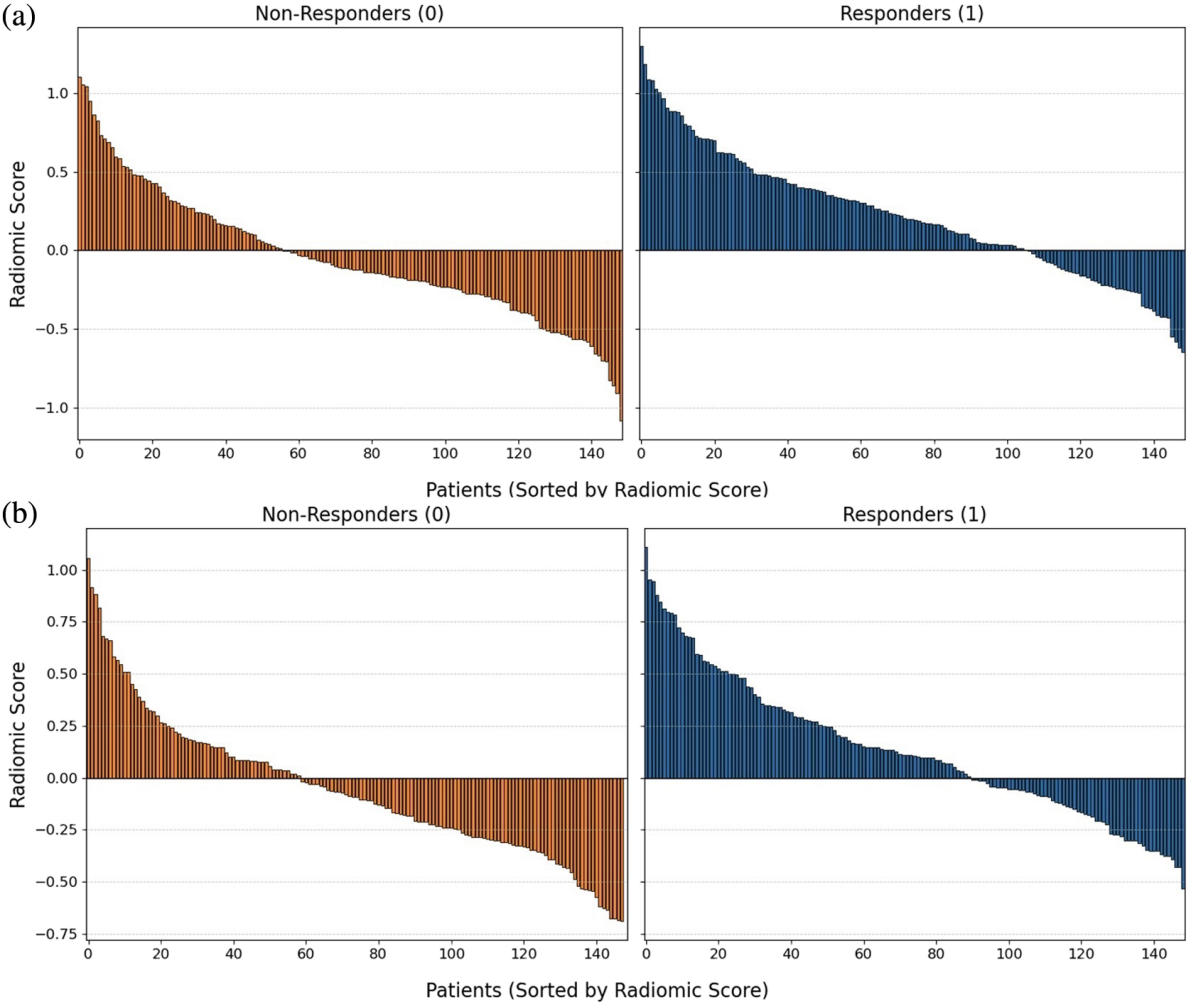
Waterfall plots of radiomic scores for (a) all tumors ROI and (b) the largest tumor ROI.

Figure 6 shows the radiomic scores analysis derived from LR models for both ROIs. Panel (a) presents waterfall plots of radiomic scores computed across all tumor ROIs, whereas panel (b) displays scores derived from the largest tumor ROI. Bars are coded by outcome (orange = non-responders [0], blue = responders [1]), whereas patients within each outcome group are ordered along the x-axis by their radiomic score. The radiomic scores of both all tumors and the largest tumor show a similar trend, where responders (blue) generally cluster in the higher radiomic score ranges, whereas nonresponders (orange) dominate the lower score ranges. However, scores for all tumors demonstrate a slightly clearer distinction and broader score range than the one for the largest tumor alone.

The feature coefficients provided in Table 3 reflect the LR’s evaluation of how different radiomic characteristics influence the likelihood of the response group, with larger absolute values of the coefficients indicating a stronger contribution to the model’s prediction. When all tumors were included in the ROI, 12 features were linked to the response group and 8 to the no-response group. For the largest tumor, seven features were associated with the response group, and four with the no-response group.

**Table 3:** Importance of radiomic features for all tumors and the largest tumor for each response group derived from LR. Features are sorted based on the magnitude of the influence on the prediction. Positive coefficients are associated with response, whereas negative values are linked to nonresponders.

ROI	Response group	Feature	Coefficient
All tumors	Response	Kurtosis	0.253009
		Maximum probability	0.227787
		Size zone nonuniformity normalized	0.213722
		Large dependence high gray level emphasis	0.201046
		Median	0.139298
		Elongation	0.126927
		Major axis length	0.096691
		Small dependence high gray level emphasis	0.065697
		Low gray level zone emphasis	0.035498
		IMC1	0.033058
		Small dependence low gray level emphasis	0.022061
		MCC	0.017350
	No response	Sphericity	−0.197938
		Robust mean absolute deviation	−0.195099
		Low gray level emphasis	−0.119958
		Cluster shade	−0.109195
		Low gray level run emphasis	−0.106471
		Contrast	−0.093332
		Energy	−0.072821
		Gray level nonuniformity normalized	−0.000407
Largest tumor	Response	Kurtosis	0.36812
		10th percentile	0.178058
		Maximum probability	0.147401
		Small area emphasis	0.086769
		Joint energy	0.068232
		Mean	0.064039
		IMC1	0.026897
	No response	Low gray level emphasis	−0.110537
		Flatness	−0.045218
		Short run low gray level emphasis	−0.035002
		Total energy	−0.017578

Kurtosis consistently demonstrated a strong positive relationship with the response group in both experiments, indicating that higher kurtosis values are associated with an increased likelihood of response. Another feature linked with response in both ROIs, although to a lesser extent, is IMC1, which quantifies the correlation of gray levels in an image. On the other hand, higher sphericity was a strong indicator of no response in all tumor experiments. In addition, low gray level emphasis, which measures how much of the image consists of dark regions, showed a moderate association with no response for both cases.

## 4 Discussion and Conclusion

Over the past few decades, medical imaging has transformed patient care by providing clearer, more detailed views of internal structures within the human body. These images are more than just visual representations; they are a source of very valuable data that is not perceptible to our human eyes. Radiomics is used to systematically extract quantitative features from these images to aid in diagnosis, prognosis, and treatment planning. The application of radiomic features to various cancer types and modalities has shown promising potential.^28^ This study demonstrates the potential of radiomics and ML to predict treatment response in unresectable CRLM patients, limiting the analysis to baseline scans to mitigate patient exposure to the toxic effects of chemotherapy. We defined to separate ROIs and conducted hand-crafted feature extraction from each of the ROI, which were further used to train ML models.

In our study, radiomics demonstrated the ability to capture stronger signals associated with treatment outcomes. The findings underscore the utility of certain features in capturing patterns associated with treatment outcomes, reflected in the predictive performance of the models. The relatively high AUCs achieved across the models demonstrate predictive accuracy.

The discovery of interpretable biomarkers linking imaging to biology identified 20 radiomic features (e.g., texture, shape) strongly associated with chemotherapy response/nonresponse, offering mechanistic insights into tumor microenvironment dynamics and potential therapeutic targets. Furthermore, the dual-ROI strategy, which involved analyzing both the entire tumor burden and the dominant tumor, demonstrated the prognostic role of tumor heterogeneity. By capturing spatially distinct prognostic signals from both all liver lesions and the largest tumor, this approach enhances model robustness and improves clinical generalizability. Our findings show that including all tumors in the analysis, rather than focusing only on the largest tumor, leads to better model performance. This suggests that capturing a more comprehensive view of tumor heterogeneity can provide stronger predictive power. However, a notable overlap in the radiomic features selected for both ROIs was observed, with 11 out of 20 features consistently identified as relevant for predicting treatment response. This suggests that these features may capture fundamental patterns related to tumor biology that remain consistent across tumors.

NB achieved the highest recall (0.799) with an AUC of 0.757, making it particularly useful in situations where identifying responders is a priority. However, this came at the cost of lower specificity, leading to a higher likelihood of misclassifying a nonresponder as a responder. Among the classifiers, the SC model stood out as the best overall performer when analyzing all tumors. This ensemble model, which integrates multiple ML models, achieved superior predictive performance (AUC: 77%) compared with single-model approaches, with enhanced reliability for clinical translation.

In contrast to our current work, which leverages a larger cohort to strengthen its findings, previous studies have examined CT-based radiomics for CRLM response prediction using comparatively small samples. Rabe et al.^29^ investigated the potential of CT texture analysis for predicting chemotherapy response in CRLM using RECIST for a small cohort of 29 nonnecrotic CRLM patients. Radiomic features were extracted from 1.25 mm CT scans, and feature selection was performed to address correlations, retaining two features (minimum histogram gradient intensity and long-run low gray level emphasis) in the final model, which achieved an AUC of 0.80%. Furthermore, Nakanishi et al.^30^ developed a radiomics-based model to predict the response of CRLM to first-line chemotherapy using CT scans, assessed according to RECIST. The study included 42 patients, with radiomic features extracted from 126 CRLMs. Feature selection through Lasso regression identified 11 key features to conduct a radiomic signature that showed strong predictive power with an AUC reaching 0.78 on the validation cohort.

Several studies have focused primarily on texture features extracted from CT and MRI to predict treatment response. Beckers et al.^31^ found a subtle, nonsignificant trend toward higher entropy in liver metastases among responders compared with nonresponders based on RECIST, suggesting that texture features such as entropy may reflect underlying changes in tumor biology that are not detectable by RECIST measurements alone. Rao et al.^32^ compared CT texture analysis with traditional RECIST-based size measurements and volumetric assessments for evaluating CRLM response. The analysis found that changes in CT texture features, particularly entropy and uniformity, significantly predicted pathological response, unlike size and volume changes. Good responders showed increased uniformity and decreased entropy from pre-treatment to post-treatment scans, reflecting a more homogeneous tissue structure after effective treatment. By contrast, Ahn et al.^33^ did not detect significant differences in baseline entropy between responders and nonresponders to chemotherapy. However, the analysis, which incorporated logistic regression and RECIST-based patient classification, identified lower 2D skewness and narrower 3D standard deviation as strong predictors for the CRLM response group. The study also performed a variation analysis across different CT scanners, finding no significant inter-scanner differences in these predictive features, supporting their robustness for clinical use.

In conclusion, this study highlights the importance of accurate prediction tools in the management of CRLM. The findings indicate a significant signal in pre-treatment imaging for predicting response, demonstrating the potential of advanced imaging and modelling techniques in optimizing treatment strategies. These methods offer a promising pathway toward personalized treatment strategies by effectively identifying the likelihood of treatment response prior to treatment.

A limitation of our study is the considerable range of imaging parameters used across the cohort, which results from the fact that the imaging data were acquired between 2009 and 2020. Technological improvements over that time have led to substantial changes in the CT scanners used, including major decreases in detector width (1.25 to 0.6 mm), and increases in detector count (4 to 128). Imaging protocols are modified over time, as new equipment is introduced, and treatment regimes evolve. As a retrospective study, these differences are unavoidable. However, a retrospective study with identical imaging protocols would severely limit the sample size from which to build prediction models. Furthermore, these variations reflect real-world differences that may better mimic variations across different imaging centers and potentially improve the generalizability of our results. Ultimately, validation across additional centers would be the best approach to show the generalizability of our results, an approach that we are currently investigating.

## Data Availability

Radiomic features extraction from CT images was done using the PyRadiomics library in Python.^34^ Code for the mRMR algorithm was adopted from github.com/smazzanti/mrmr open-source repository. All ML models used in this study were implemented using the scikit-learn^25^ library. To protect patient privacy and uphold ethical standards, the original imaging data cannot be shared publicly. However, the extracted radiomics features are available upon reasonable request.
